# Vorticity for the assessment of pulmonary vascular hemodynamics in pulmonary arterial hypertension

**DOI:** 10.1186/1532-429X-16-S1-P15

**Published:** 2014-01-16

**Authors:** Alexander Honeyman, James Browning, Jean Hertzberg, Joyce D Schroeder, Aurelien F Stalder, J Kern Buckner, Brett Fenster

**Affiliations:** 1 Division of Cardiology, National Jewish Health, Denver, Colorado, USA; 2 Mechanical Engineering, University of Colorado, Boulder, Colorado, USA; 3 Division of Radiology, National Jewish Health, Denver, Colorado, USA; 4 Magnetic Resonance, Imaging and Therapy, Healthcare Sector, Siemens AG, Erlangen, Germany

## Background

4D flow CMR analysis of main pulmonary artery (MPA) flow in pulmonary arterial hypertension (PAH) has demonstrated vortical formations whose existence time correlates with mean pulmonary arterial pressure (MPAP). Vorticity can quantitate the rotation of these vortices and may represent a novel way to assess pulmonary arterial hemodynamics. We aimed to determine if MPA vorticity correlates with pulmonary vascular hemodynamics in PAH subjects when compared to controls using 4D flow CMR.

## Methods

Seven PAH subjects (5 females/2 males) and 4 age-matched controls (3 females/1 male) underwent same-day right heart catheterization (RHC) and 4D flow CMR. MPAP, pulmonary vascular resistance (PVR), pulmonary capillary wedge pressure (PCWP), and stroke volume (SV) were measured during RHC. Pulmonary vascular compliance (Ca) was calculated by dividing SV by pulmonary pulse pressure. 4D flow CMR was performed with interleaved 3-directional velocity encoding (spatial resolution = 3.5 × 2.6 × 3.0 mm 3, α = 15°, TE/TR = 2.85/48.56 ms, venc = 150 cm/s, temporal resolution = 50 ms) on a 1.5 T MRI system (Avanto, Siemens, Germany) using ECG gating and respiratory navigation. Images were acquired in a sagittal oblique 3D volume covering the entire right heart and MPA. Datasets were corrected for noise (Jelena Bock, Northwestern University) and aliasing using a custom Matlab program. Corrected data was imported into Paraview (Kitware, Clifton, NY) for isolation of the MPA. MPA peak systolic vorticity was then calculated using Paraview. Univariate regression analysis was used to test the relationship between peak systolic vorticity and pulmonary hemodynamics using JMP (SAS, Cary, NC).

## Results

No significant difference in age, gender, or body mass index existed between the control and PAH groups. When PAH subjects were compared to controls, MPAP was significantly elevated (42 ± 15 vs.22 ± 1 mmHg, p 0.02), compliance was significantly reduced (1.4 ± 0.8 cc/mmHg vs. 4.0 ± 1.8 cc/mmHg, p < 0.01), and PVR was elevated (11 ± 9 vs. 2 ± 1 Woods units, p 0.05). MPA vorticity was significantly reduced in PAH subjects (3904 ± 3254 vs. 10070 ± 7585 1/s) and correlated with all indices of afterload including Ca (r 0.92, p < .001), MPAP (r 0.63, p 0.04), as well as PVR (r 0.62, p 0.04).

## Conclusions

In a small cohort, MPA peak systolic vorticity was reduced in PAH subjects and correlated with all invasively-measured pulmonary vascular hemodynamics. 4D CMR-derived MPA vorticity may represent a novel research tool to the study the role of complex blood flow formations in the pathogenesis and hemodynamic progression of PAH.

## Funding

Siemens Medical Solutions.

